# Broadcast Spawning Coral *Mussismilia hispida* Can Vertically Transfer its Associated Bacterial Core

**DOI:** 10.3389/fmicb.2017.00176

**Published:** 2017-02-07

**Authors:** Deborah C. A. Leite, Pedro Leão, Amana G. Garrido, Ulysses Lins, Henrique F. Santos, Débora O. Pires, Clovis B. Castro, Jan D. van Elsas, Carla Zilberberg, Alexandre S. Rosado, Raquel S. Peixoto

**Affiliations:** ^1^Institute of Microbiology, Federal University of Rio de JaneiroRio de Janeiro, Brazil; ^2^Institute of Biology, Federal University of Rio de JaneiroRio de Janeiro, Brazil; ^3^Instituto Coral VivoSanta Cruz Cabrália, Brazil; ^4^National Museum, Federal University of Rio de JaneiroRio de Janeiro, Brazil; ^5^Department of Microbial Ecology, Centre for Ecological and Evolutionary Studies, University of GroningenGroningen, Netherlands

**Keywords:** holobiont, microbiota transmission, *Symbiodinium*, coral core microbiome, bacteria

## Abstract

The hologenome theory of evolution (HTE), which is under fierce debate, presupposes that parts of the microbiome are transmitted from one generation to the next [vertical transmission (VT)], which may also influence the evolution of the holobiont. Even though bacteria have previously been described in early life stages of corals, these early life stages (larvae) could have been inoculated in the water and not inside the parental colony (through gametes) carrying the parental microbiome. How *Symbiodinium* is transmitted to offspring is also not clear, as only one study has described this mechanism in spawners. All other studies refer to incubators. To explore the VT hypothesis and the key components being transferred, colonies of the broadcast spawner species *Mussismilia hispida* were kept in nurseries until spawning. Gamete bundles, larvae and adult corals were analyzed to identify their associated microbiota with respect to composition and location. *Symbiodinium* and bacteria were detected by sequencing in gametes and coral planula larvae. However, no cells were detected using microscopy at the gamete stage, which could be related to the absence of those cells inside the oocytes/dispersed in the mucus or to a low resolution of our approach. A preliminary survey of *Symbiodinium* diversity indicated that parental colonies harbored *Symbiodinium* clades B, C and G, whereas only clade B was found in oocytes and planula larvae [5 days after fertilization (a.f.)]. The core bacterial populations found in the bundles, planula larvae and parental colonies were identified as members of the genera *Burkholderia, Pseudomonas, Acinetobacter, Ralstonia, Inquilinus* and *Bacillus*, suggesting that these populations could be vertically transferred through the mucus. The collective data suggest that spawner corals, such as *M. hispida*, can transmit *Symbiodinium* cells and the bacterial core to their offspring by a coral gamete (and that this gamete, with its bacterial load, is released into the water), supporting the HTE. However, more data are required to indicate the stability of the transmitted populations to indicate whether the holobiont can be considered a unit of natural selection or a symbiotic assemblage of independently evolving organisms.

## Introduction

The hologenome theory of evolution (HTE) considers the host and its associated microbiota genomes combined as one unit of natural selection in evolution (Rosenberg et al., 2007; Zilber-Rosenberg and Rosenberg, 2008). However, different aspects regarding the evolution of the genomes of hosts and their symbionts, as well as complex aspects of the evolutionary processes, remain under discussion (Leggat et al., 2007; Rosenberg et al., 2007; Moran and Sloan, 2015). In fact, assumptions underpinning this theory that the microbiome, or parts thereof, can be transmitted from one generation to the next and may influence the evolution of the coral holobiont have not been completely elucidated and remain under debate.

Corals depend on their associated microbiome (Rosenberg et al., 2007). Previous studies have indicated that the majority of broadcast spawning coral species do not vertically transfer their microalgal symbionts (*Symbiodinium* spp. Freudenthal, 196) (Freudenthal, 1962; Knowlton and Rohwer, 2003; Fabina et al., 2012) and other microbial symbionts (Sharp et al., 2010) via their gametes. They generally acquire symbionts as larvae or during post-settlement and metamorphosis as a juvenile polyp via horizontal transmission (from seawater) (Weis et al., 2001; Knowlton and Rohwer, 2003; Apprill et al., 2009). However, new data have reported the vertical transmission (VT) (from a parental colony) in broadcast spawning coral *Montipora capitata*, which transfers its *Symbiodinium* through its eggs (Padilla-Gamiño et al., 2012). Many studies have also confirmed that brooded coral transmit their *Symbiodinium* vertically from parent to offspring through their larvae (Fadlallah, 1983; Harrison and Wallace, 1990; Hirose et al., 2001; Thornhill et al., 2006). Less information is available about the transmission of other important coral microbial symbionts, such as bacteria. Sharp et al. (2011) demonstrated that *Roseobacter* spp. are vertically translocated from the parental colony to newly released larvae in the brooded coral *Porites astreoides*. On the other hand, some larvae seem to remain free of bacteria after fertilization, being capable of acquiring bacteria directly from the seawater during larval or polypoid stages (Apprill et al., 2009). Moreover, horizontal transmission of coral-associated bacteria in seven broadcast spawning coral species was reported by Sharp et al. (2010). Littman et al. (2009) and Lema et al. (2014) have also reported changes and uptake of bacteria in early stages of corals but not inside the oocytes.

Considering the controversial data regarding *Symbiodinium* transmission and the absolute lack of information concerning bacterial core diversity, dynamics and transmission in broadcast spawning corals, specifically the lack of data on this transmission through coral gametes, we here investigate and identify the (core) bacteria and *Symbiodinium* associated with different life stages of the Brazilian endemic coral *Mussismilia hispida* (Verrill, 1902). *M. hispida* is a hermaphrodite species that releases gametes in the water column in a seasonal spawning event that occurs over three consecutive months (Pires et al., 1999; Pires et al., 2016). Approximately 1/3 of the stony coral reef fauna of Brazil are endemic, and most are major builders of Brazilian reefs, including the *Mussismilia* genus.

## Materials and Methods

### Ethics Approval and Consent to Participate

Permission for sampling was obtained from the Brazilian Institute of Environment and Renewable Natural Resources (IBAMA)/Chico Mendes Institute for Biodiversity Conservation (ICMBio), permanent permission number 16942, in accordance with the Instruction Normative n° 03/2014 of System Authorization and Information on Biodiversity (SISBIO) and from local authorities of the Municipality Environmental Agency (SMMA)/Porto Seguro, Bahia, Brazil.

### Spawning Event and Sampling Procedures

Approximately, seven *M. hispida* colonies were collected between latitudes 16°23′30″ S and 16°25′06″ S and longitudes 038°58′30″ W and 038°59′18″ W approximately 1 month (August, 2012) before the spawning event at “Recife de Fora,” Porto Seguro, Bahia, Brazil. These colonies were kept in tanks during the experiment. Seawater was collected directly from the beach and kept in a reservoir that distributed water to the tanks. Tanks had a 1000 L (1.1 m diameter) capacity and were kept in a room at 25°C with aerators to maintain oxygenation and circulation of the water. After this period, approximately 50% of the water was changed daily.

A spawning event occurred naturally on September 2012 (Pires et al., 2016). Some gamete bundles (approximately 15–20) of three coral colonies were collected immediately after spawning and kept at -20°C, while others were transferred to three different aquaria. Gamete fertilization occurred naturally inside each aquarium. All aquaria had an 80 L (40 cm × 40 cm × 50 cm) capacity and were kept in a room at 25°C with aerators to maintain oxygenation and circulation of the water. In the first 24–48 h, the water was not changed to minimize loss of sperm. After this period, approximately 50% of the water in each aquarium was changed daily. Corals, gametes, and coral planula were still exposed to natural day–night cycles. Approximately 25 coral planula larvae (3 and 5 days after fertilization) from the three coral colonies were collected, washed with sterile NaCl (3%) to minimize microbial contamination from seawater (to perform sequencing analysis) and kept at -20°C. Immediately after spawning, *M. hispida* fragments (approximately 30 mm diameter) from the three different colonies that released the bundles were also collected and kept at -20°C. Surrounding seawater samples were also collected from the tanks and aquaria during the spawning event and 3 and 5 days after spawning. In each case, approximately 1000 mL of seawater was filtered through a 0.22 μm filter. Sampling was performed in triplicate so that each colony was a replica, with a total of 21 samples. All samples were frozen in -80°C for molecular analyses. A summary of the experimental design that was used in this study can be seen in **Supplementary Figure S1**.

### Light and Transmission Electron Microscopy

For light and transmission electron microscopy (TEM), embryonic and coral planula larvae stages coral (three samples from each bundle and planula larvae sample) were fixed in 4% formaldehyde and 2.5% glutaraldehyde, respectively, in 0.1 M cacodylate buffer pH 7.3 prepared with deionized water. To preserve the structure of *M. hispida* bundles, they were immobilized with agarose solution 3.5% (v/v). Then, the samples were post-fixed in buffered 1% OsO_4_ for 2 h, washed in the same buffer, dehydrated in an acetone series (30, 50, 70, 90, and 100% 3x), embedded in Polybed resin, and polymerized for 72 h at 60°C.

Thin (2 μm) and ultra-thin (70 nm) sections were obtained in a Leica ultramicrotome (Leica Microsystems, Vienna, Austria). Thin sections were stained toluidine blue (1%) and imaged with a Zeiss Axioimager (Carl Zeiss, Jena, Germany) optical light microscope. Ultra-thin sections were placed in a copper grids, stained with uranyl acetate and lead citrate and imaged with an FEI Morgagni (FEI Company, Eindhoven, The Netherlands) TEM operating at 80 kV.

### DNA Extraction

Coral samples were crushed and homogenized (3 polyp fragments from adult corals, approximately 15–20 bundles from each coral colony, and approximately 25 coral planula larvae, at 3 and 5 days a.f, composed samples from the three coral colonies) and were collected from each coral sample. The DNA (water and coral samples) was extracted using the PowerSoil^®^ DNA Isolation Kit (MoBio Laboratories, Carlsbad, CA, USA) following a modification of the method described by Sunagawa et al. (2010).

### *Symbiodinium* Diversity

The amplification of a specific region of the 28S rRNA gene of *Symbiodinium* was performed using the primers D1D2-F and D1D2-R (Loh et al., 2001) in a solution containing 1X PCR buffer, 0.2 mM dNTPs, 2.5 mM MgCl_2_, 2.5 U of recombinant *Taq* DNA polymerase (Promega, Madison, WI, USA), 10–40 ng of total DNA, 0.5 μM of each primer and sterile Milli-Q water in a final volume of 15 μl. The reaction was performed in a thermocycler (Mastercycler Gradient, Eppendorf, Hamburg, Germany) with the following conditions: initial denaturing at 94°C for 5 min; 1 cycle at 94°C for 1 min, 56°C for 45 s and 72°C; 29 cycles at 92°C for 1 min, 56°C for 1 min and 72°C for 45 s; and a final extension at 72°C for 10 min.

Polymerase chain reaction products were cloned using the pJET 1.2 vector (CloneJET PCR Cloning Kit #K1231, Thermo Scientific) and transformed into *Escherichia coli* competent cells (Single Step – KRX – Competent Cells, Promega) with the TransformAid Bacterial Transformation Kit (#K2710 Thermo Scientific) according to the manufacturer’s instructions. Approximately 50 clones were re-amplified for the 28S rRNA gene fragments and digested using the restriction enzyme *Taq* I (Promega, cutting site 5′T/CGA3′) to perform a screening analysis using RFLPs (Restriction Fragment Length Polymorphisms). The RFLPs enabled the selection of distinct *Symbiodinium* lineages for sequencing. Digestions were performed in a solution containing the enzyme buffer 1X, 4 μl of the PCR product, 5 U of *Taq* I and sterile Milli-Q water in a final volume of 20 μl. Reactions were incubated at 65°C for approximately 16 h. All digestion products were visualized by agarose gel electrophoresis 2% (v/v) in 0.5X TBE buffer, stained with ethidium bromide and visualized under UV light. *Symbiodinium* clades were identified by comparisons with RFLP patterns of pure cultures of *Symbiodinium* that commonly occur in the Atlantic (clades A, B, C, and D), obtained by donation from Prof. Mark Warner, University of Delaware, USA.

After selection by RFLP, clones from each clade were amplified and purified by the automatic robot epMotion 5075 (Eppendorf, Hamburg, Germany) and directly sequenced by Sanger method (Sanger et al., 1977) in the automatic sequencer ABI 3500 (Applied Biosystems). Sequences were manually trimmed removing the low quality ends and primers using the SeqMan 7 software (DNAStar, Inc.). Sequences with a final length of 601 bp were obtained. All sequences were deposited in the GenBank database with the accession number (KX459520-KX459562).

### Bacterial Profile

Specific regions of the gene encoding the 16S rRNA gene were amplified using the primers U968*f* GC and L1401*r* (Heuer and Smalla, 1997) in a solution containing 1X PCR buffer, 0.2 mM dNTPs, 2.5 mM MgCl_2_, 2.5 U of recombinant Taq DNA polymerase (Promega, Madison, WI, USA), 10–40 ng of total DNA, 200 μmol of each primer and sterile Milli-Q water in a final volume of 50 μl. The reaction was performed in a thermocycler (Mastercycler Gradient, Eppendorf, Hamburg, Germany) with the following conditions: initial denaturing at 94°C for 3 min; 35 cycles at 94°C for 1 min, 55°C for 1 min and 72°C for 1 min; and a final extension at 72°C for 10 min. The denaturing gradient gel elecrtophoresis (DGGE) gels (45–65% urea and formamide) were prepared with a solution of polyacrylamide (6%) in Tris-acetate (pH 8.3). Electrophoresis was performed in Tris-acetate-EDTA buffer at 60°C at a constant voltage of 75 V for 16 h. The DGGE gels were stained with SYBR Green (Invitrogen, Carlsbad, CA, USA) and visualized using a Storm 860 Imaging System (GE Healthcare, Milwaukee, WI, USA).

### Ion Torrent PGM Sequencing

Based on the obtained bacterial profiles, samples with different profiles were selected to perform the sequencing approach. The 16S rRNA gene V4 variable region PCR primers 515F/806R (Caporaso et al., 2011) were used in a single-step 30 cycle PCR using the HotStarTaq Plus Master Mix Kit (Qiagen, USA) under the following conditions: 94°C for 3 min, followed by 28 cycles (five cycle used on PCR products) of 94°C for 30 s, 53°C for 40 s and 72°C for 1 min, followed by a final elongation step at 72°C for 5 min. Sequencing was performed at MR DNA (www.mrdnalab.com, Shallowater, TX, USA) in an Ion Torrent PGM following the manufacturer’s guidelines.

The QIIME (Caporaso et al., 2010b) software package was used to process the raw sequence data. In brief, sequences were trimmed using the following parameters: quality score > 25, sequence length > 200, maximum length of homopolymer of 6 and 0 mismatched bases in the primers and barcodes.

The remaining high-quality sequences were binned into operational taxonomic units (OTUs) at 97% sequence identity USEARCH 6.1 methodology (version v6.1.544) followed by selection of a representative sequence. Chimeric sequences were also identified using USEARCH 6.1 methodology (version v6.1.544) (Edgar, 2010) and removed. A representative sequence for each phylotype was aligned against the Greengenes database (Desantis et al., 2006) using PyNAST (Caporaso et al., 2010a), with sequences classified using the Greengenes taxonomy via RDP classifier (Wang et al., 2007). Before further analysis, singletons and chloroplast plastid, mitochondrial and archaeal sequences were removed from the dataset. For all OTU-based analyses, the original OTU table was rarified to a depth of 14500 sequences per sample to minimize the effects of sampling effort on the analysis. The QIIME package was also used to generate weighted UniFrac distance matrices (Lozupone et al., 2006) and α-diversity metrics, including richness and diversity indices. All sequences were deposited in the NCBI Sequence Read Archive database with the accession numbers (SRR3740771–SRR3740793).

The core microbiome of gametes, planula larval, and parental coral colony was identified using QIIME and determined by plotting OTU abundance. The coral bacterial core was represented by OTUs that were shared by 100% of the samples.

### Statistical Analyses

The DGGE band profiles were digitalized and converted into data matrices using the Bionumerics v6.0 package (Applied Maths) according to the manufacturer’s instructions. The matrices were ordered by NMS (Kruskal, 1964; Mather, 1976) using a Bray–Curtis distance matrix. To analyze the difference between profiles and composition of bacterial communities, we used non-metric scaling (NMS) with Past 3.x Software (Hammer et al., 2001). To assess the variation among different samples (parental colony, bundles and larvae), we used a permutational multivariate analysis of variance (PERMANOVA; Kelly et al., 2015) using Past 3.x Software (Hammer et al., 2001).

*Symbiodinium* diversity was assessed using the resulting 28S rRNA gene sequences and performing searches using the *Basic Local Alignment Search Tool* – BLAST tool^1^ (Altschul et al., 1990). Additionally, phylogenetic reconstruction analysis was performed using the 28S rRNA gene sequences from this study together with representatives of each of the *Symbiodinium* clade that were found by BLAST searches (downloaded from the NCBI database). Sequence alignments were performed using MEGA 5 software (Tamura et al., 2011) with the ClustalW tool (Larkin et al., 2007), and phylogenetic analysis was conducted with the Maximum-Likelihood method (ML) recovered in the PhyML platform (Guindon et al., 2010). The phylogeny were reconstructed with the substitution model GTR+I+G [General Time Reversible with Invariable sites proportion fixed and Gamma-distributed rates (Buckley et al., 2001)], and phylogenetic clade support was calculated by the 1000 bootstrap resampling method. ML trees were edited using FigTree 1.4.2 software^2^.

## Results

### Bacterial Communities in Different Life Stages of *M. hispida*

**FIGURE 1:**
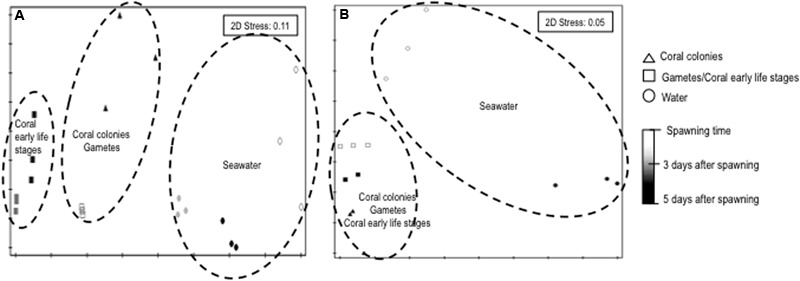
**NMS (Bray–Curtis) plot of bacterial communities associated to different life stages of *Mussismilia hispida* and surrounding seawater based on PCR-DGGE (A)** and high throughput sequencing **(B**) data (*n* = 3). Contours are based on significant pairwise PERMANOVA results (*p* = 0.01).

The 16S rRNA gene-based PCR-DGGE and next-generation sequencing analyses of the bacterial assemblages associated with three parental coral colonies, bundles (gametes) and planula larvae stages and seawater samples revealed a clear clustering of patterns according to the sample. Non-metric dimensional scaling (NMDS) of the data clearly indicated a dichotomy in the clustering, with one major cluster being subdivided in two subclusters (**Figures 1A,B**). The patterns derived from the gametes (bundles) and representatives of the coral planula larvae stages were closely related to those from the parental coral, in contrast to the seawater samples, which clustered apart. PERMANOVA confirmed the results of the NMDS analysis, demonstrating that the bacterial assemblages varied significantly among the parental coral, bundles, coral planula larvae stages and water-derived patterns (*p* = 0.01; Pseudo-*F* = 9.46 and 8.94, respectively) (**Figures 1A,B**). The results from the pairwise PERMANOVA suggested that the microbial communities of the bundles and coral planula larvae stages were significantly related to those from the parental coral microbial communities (**Supplementary Table S1**). As the microbial community structure of coral planula larvae (3 and 5 days a.f.) were very similar, only coral planula larvae 5 days a.f. were used for sequencing (**Figure 1A**). Bacterial community richness decreases in coral and water samples between spawning time and after 5 days after spawning (**Supplementary Figure S2**).

### *Symbiodinium* Diversity at Different Life Stages of *M. hispida*

**FIGURE 2:**
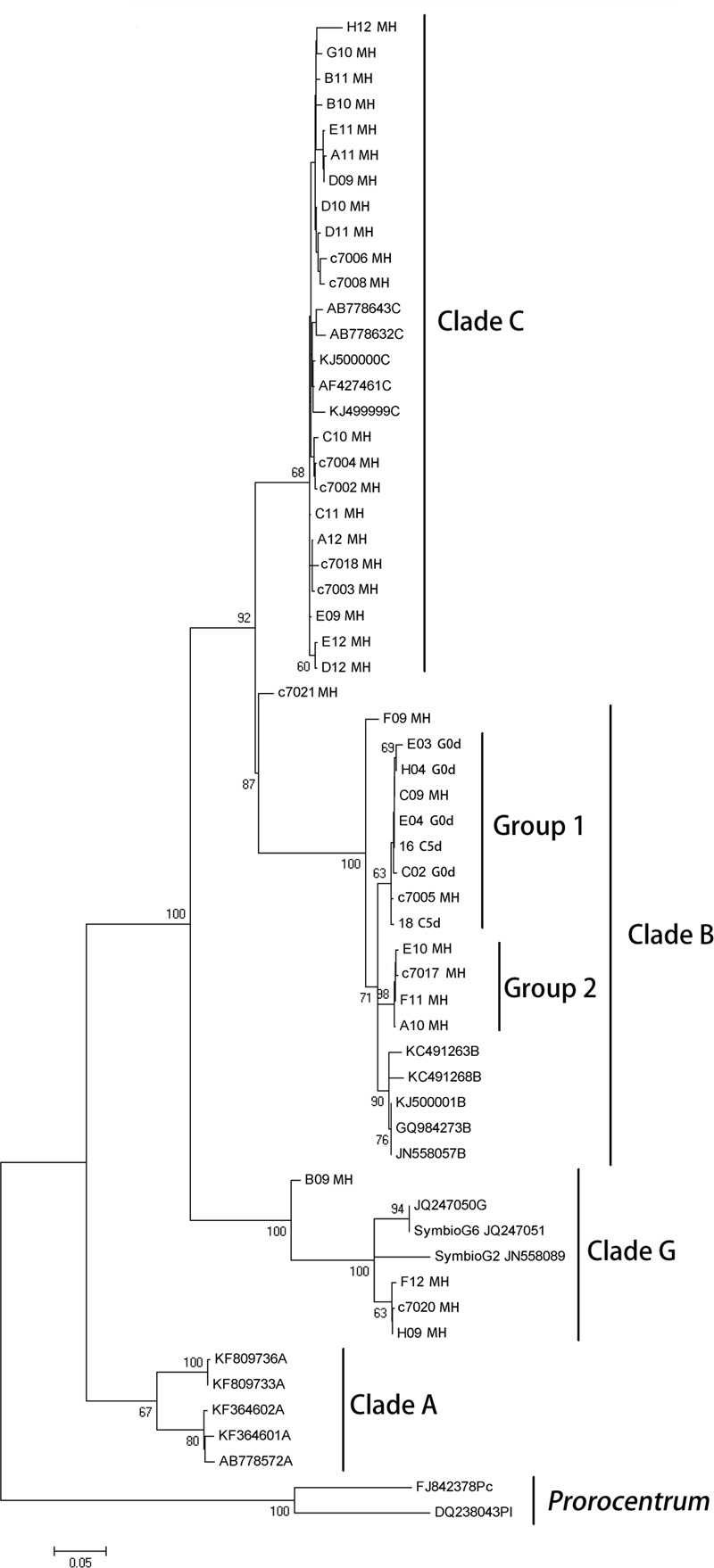
**Maximum likelihood phylogenetic reconstruction of *Symbiodinium* inferred from partial 28S rRNA gene sequences, including sequences obtained from *M. hispida* parental colony, gametes and planula larvae stages and sequences downloaded from the NCBI database (see GenBank accession numbers in Supplementary Material).** Sequences from *Prorocentrum* spp. was used as outgroup. G0d: bundles at the spawning event, L5d: coral planula larvae, 5 days a.f., and MH: adult colonies of *M. hispida*. Bootstrap support values are indicated at the base of branches.

The phylogenetic reconstruction analysis of the 28S region detected clades B, C, and G in the parental colony of *M. hispida*, while only clade B was detected in gamete bundles and representatives of the coral planula larvae stages (**Figure 2**). Additionally, the parental colony seemed to harbor three distinct lineages of B that differ from the sequences downloaded from the NCBI database. The phylogenetic analysis also showed that the bundle and coral planula larvae harbor only one of the lineages (clade B) found in the parental colony (group 1 in **Figure 2**).

### Core Microbiome at Different Life Stages of *M. hispida*

**FIGURE 3:**
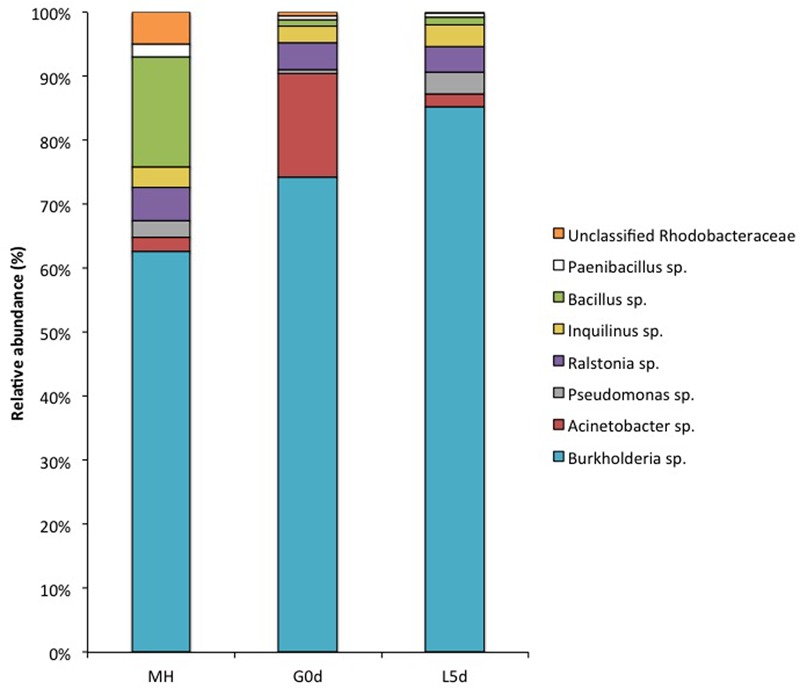
**Relative abundance of the core microbiome genera from different *M. hispida* life stages provided by 16S rRNA gene high throughput (Ion Torrent PGM) sequencing data.** G0d: bundles at the spawning event, L5d: coral planula larvae, 5 days a.f., and MH: adult colonies of *M. hispida*.

The coral bacterial “core” (shared microorganisms among all samples) represented 10.5% of the total microbial diversity in coral adult colonies and 60.7% in bundles stage and increased to 84.7% in coral planula larvae 5 days a.f. (**Supplementary Figure S3**). The genera *Burkholderia, Pseudomonas, Acinetobacter, Ralstonia, Inquilinus*, and *Bacillus* composed the coral bacterial “core” at different life stages of *M. hispida* (**Figure 3**; **Supplementary Table S2**). *Burkholderia* was the most abundant genus associated with the early stages of this coral life, representing approximately 75% of the gamete microbiome and increasing by 10% in coral planula larvae 5 days a.f. On the other hand, the relative abundance of *Acinetobacter* spp. decreased from 15% (gamete stage) to less than 5% (coral planula larvae 5 days a.f.). The abundance of the genera *Ralstonia* and *Inquilinus* remained stable, both at approximately 10%, from the time of spawning (in the gamete bundles) until the coral planula larvae 5 days a.f.

### Location of the Coral Symbionts

**FIGURE 4:**
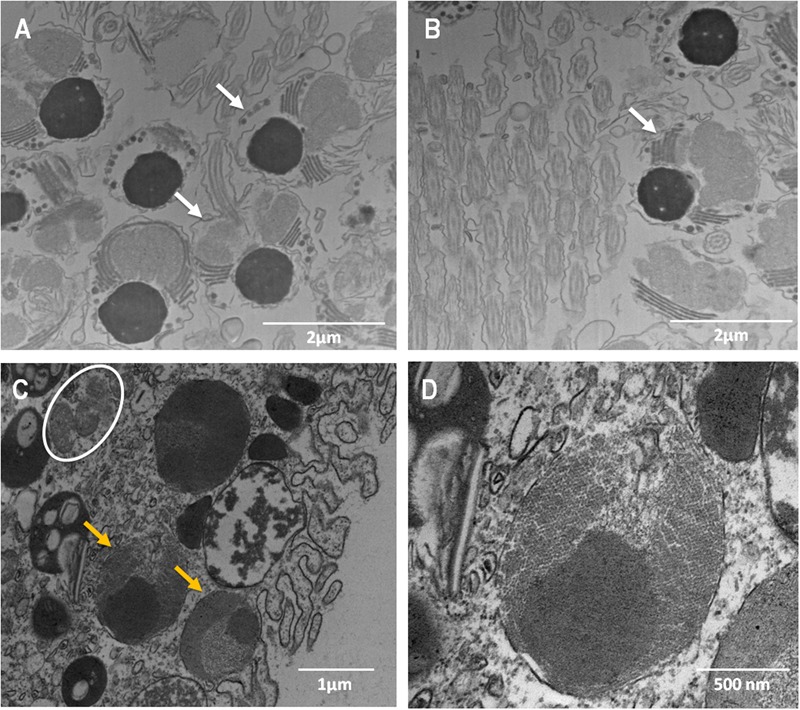
**Ultrastructure of male (A,B)** and female **(C,D)** gametes of *M. hispida*, highlighting the presumptive *Symbiodinium*-like cells in the oocytes. **(D)** Is a zoom of **(C)**. White arrow indicate the male gametes, orange arrowheads the *Symbiodinium*-like cells and white circles indicate mitochondria.

**FIGURE 5:**
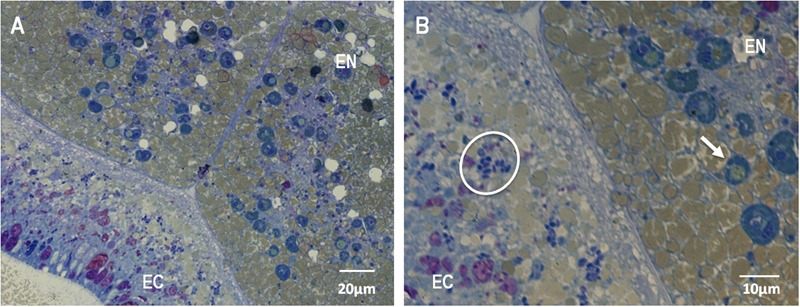
**Light microscopy micrograph of a thin section of *M. hispida* coral planula larvae (B)** is a zoom of **(A)**, 5 days a.f., highlighting the *Symbiodinium*-like cells into the endodermis **(B)**. EN: endodermis, EC: ectodermis. White arrows indicate the *Symbiodinium*-like cells. White circles indicate bacteria-like structures.

The evaluation of the bundle sections (**Supplementary Figure S4**) did not reveal the presence of *Symbiodinium* or bacterial cells. However, cells identified as *Symbiodinium-like cells* but that were smaller than *Symbiodinium* cells were observed in the peripheral region of the female gametes (eggs) (**Figures 4A,B**). These cells were found in the endodermis of the coral planula larvae 5 days a.f. No bacteria were observed inside the eggs (**Figures 4A,B**) or close to the spermatic cysts (**Figures 4C,D**). In the coral planula larvae stages (**Figure 5**), some bacterium-like structures were observed in the epidermis.

## Discussion

To the best of our knowledge, this is the first report that shows the bacterial core composition and dynamics at early stages of coral life, including gametes, as discussed below. We showed that the spawner coral *M. hispida* can vertically transfer its bacteria and *Symbiodinium* cells.

Considering the transmission of bacterial symbionts, there is no consensus or pattern on the transmission of these microorganisms. A study on the gametes and planulae larvae of other seven broadcast spawner coral species did not find any bacterial association in these life stages (Sharp et al., 2010). Thus, these corals may acquire their associated microbiota from the seawater (horizontal transmission). On the other hand, Ceh et al. (2012) investigated the microbiome of three different broadcast spawner and brooder corals (*Acropora tenuis, Pocillopora damicornis*, and *Tubastrea faulkneri*) before and after spawning and indicated that the bacterial diversity increased after spawning in all coral species. The authors highlighted that *Roseobacter* spp. was an important genus transmitted during coral reproduction. They also observed a decrease in some key groups that could have evolved through bacterial VT. However, the authors could not prove the occurrence of VT because only adult corals were sampled.

Here, not only adult colonies but also gametes and coral planula larvae at 3 and 5 days a.f. were sampled. The sequencing data showed that the *M. hispida* microbiome was composed by bacteria and *Symbiodinium* cells, even in gametes, although we could not verify the presence of bacterial and *Symbiodinium* cells in association with the (female and male) gametes of *M. hispida* through microscopy. Sequencing data also showed a stable core bacterial microbiome from the gametes to planula larvae stages that was also present in the parental coral colony. In addition, we verified that the stable microbiome represents a greater proportion of the associated bacteria in gametes and planula larvae stages. In contrast, the core microbiome represents a low proportion in the adult colonies, indicating microbial transmission once these microorganisms have left the parental colony for the bundles. The core microbiome presence could indicate a potential key function of this group in early life stages of coral development. Therefore, the obtained data suggest that this coral could use parallel and different strategies for microbiome acquisition. Considering our sequencing data, the bacterial core is transmitted vertically, potentially via the mucus that visually binds the gametes together in a bundle (observed *in vivo*). Most likely, the offspring can be partly colonized post-spawning through the uptake of microbial associates released by the parental colony into the surrounding seawater, as previously described (Apprill et al., 2009; Sharp et al., 2011; Ceh et al., 2012, 2013), and partly colonized by parental colonies, which transfer their bacterial core by seeding the mucus with parental-derived bacterial symbionts, as indicated by our data.

Thus, while *Symbiodinium* cells could be transmitted inside gametes, as shown in literature and suggested by our molecular data, the core bacterial community – being present in abundance within the mucus, which will form the bundle – could be vertically transmitted ‘from outside’ of the gametes, inside the coral. This does not exclude that some bacteria are eventually transmitted inside the gamete or even inside the *Symbiodinium* cells being transmitted in the eggs; we could not detect these bacteria due to poor resolving power of our methods. The presence of bacterial symbionts living in the *Symbiodinium* was previously reported by Ainsworth et al. (2015) in tissues of adult corals. On the other hand, *Symbiodinium* cells, which can be transmitted inside oocytes, can also be acquired from seawater in the planula larvae stage or even by coral adults (Boulotte et al., 2016).

**FIGURE 6:**
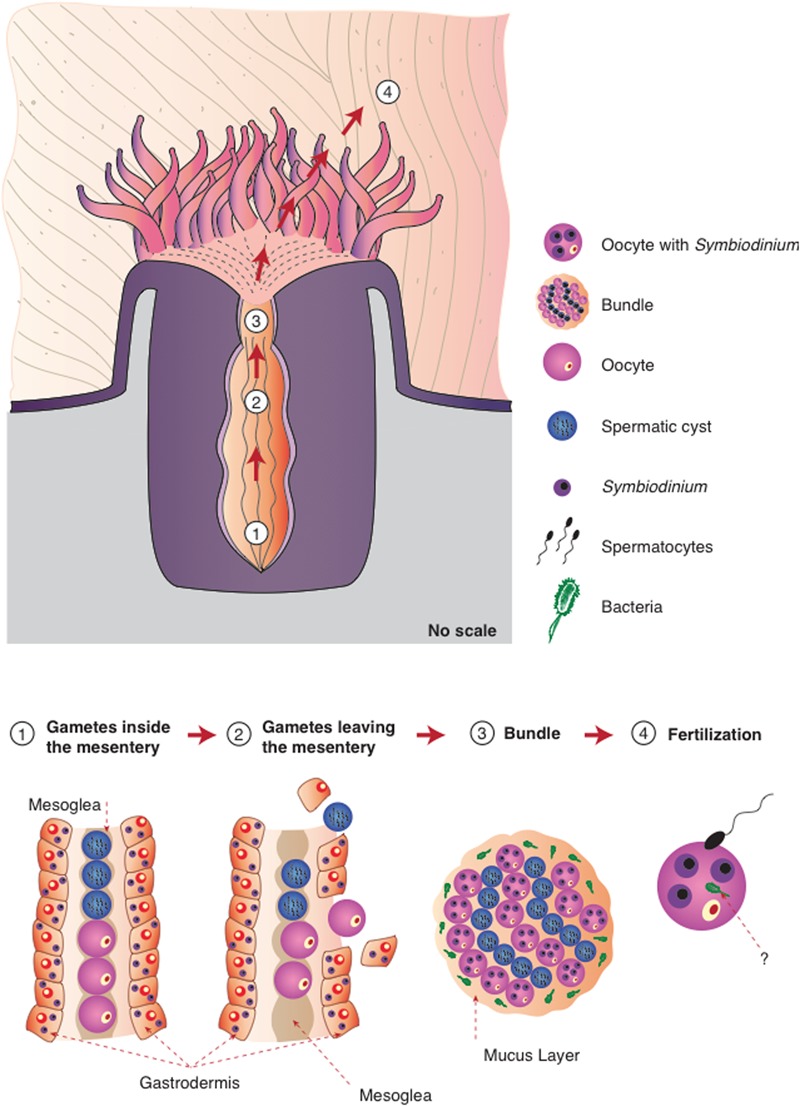
**Proposed model for the different strategies of microbiome transmission in the broadcast spawning coral *M. hispida*.** This model uses the knowledge already available in literature (Apprill et al., 2009; Sharp et al., 2011; Ceh et al., 2012, 2013) and includes our new information, providing this mural with a comprehensive view of the microbiome transmission (1) Gametes are located inside the mesentery without any symbiotic association (*Symbiodinium* or bacterial cells); (2) Gametes leave the mesentery, and *Symbiodinium* cells could be acquired by the oocytes; (3) During the bundle formation the spermatic cysts and eggs containing *Symbiodinium* cells are surrounded by the mucus full of bacterial cells; (4) Bacterial cells can be also acquired during fertilization. It is also possible that bacterial cells could be transmitted inside oocytes, or even inside *Symbiodinium* cells.

Herein, we propose the model presented in **Figure 6** for the transmission of microorganisms in this coral species. *M. hispida* is a broadcaster spawner and a hermaphroditic species; therefore, its eggs and spermatic cysts develop inside the same mesenteries, but oogenesis and spermatogenesis start at different periods, with oogenesis beginning several months before spermatogenesis (Pires et al., 1999). During gamete development and spawning, the gametes are initially in the gastrodermis, migrating to the mesoglea during the vitellogenesis period. When the eggs and spermatic cysts are mature, the rupture of the mesentery wall occurs, and the bundles that formed (eggs, cysts, and mucus) reach the gastrovascular cavity, passing through the mouth to reach the surface (Pires et al., 1999). This mural contains knowledge already available in the literature (Apprill et al., 2009; Sharp et al., 2011; Ceh et al., 2012, 2013) and includes our new data, providing this mural a very comprehensive view of coral microbiome transmission in *M. hispida*, potentially applicable to other coral species.

Regarding the preliminary survey of the dominant *Symbiodinium* clades diversity, according to Picciani-de-Souza et al. (2016), *M. hispida* colonies from Bahia, Brazil, shelter only *Symbiodinium* belonging to clade C, while possessing clades A and B at other locations along the Brazilian coast. In contrast, only clades B, C, and G were found in our parental colonies, which suggests that this coral can acquire new clades or have them in lower abundance under normal field conditions, including the rare association with clade G (commonly found in Foraminifera hosts (Pochon et al., 2001; Coffroth and Santos, 2005). However, when conditions change (i.e., aquarium confinement or pre-spawning event), the abundance of less abundant clades can be enhanced or simply detected by PCR, a technique that detects mostly dominant sequences. Additionally, although *Symbiodinium* clades are transferred vertically, clade selection seems to occurs early, since only clade B was found in the embryonic and planula larvae stages. Abrego et al. (2009) demonstrated the presence of only *Symbiodinium* of clade C in parental colonies of acroporid corals, whereas only *Symbiodinium* of clades A or D were found in the larvae, though the mechanisms behind these associations remain unknown. Our data corroborate the findings of Padilla-Gamiño et al. (2012), demonstrating that *Symbiodinium* can be transmitted vertically, even in coral species where fertilization of gametes and larval development occur in seawater. Boulotte et al. (2016) demonstrated that *Symbiodinium* cells can also be acquired from the environment by adult corals, and Hester et al. (2016) indicated the presence of a stable core microbiome comparing different coral species and locations but also demonstrated that coral–microbiome associations are not exclusive and therefore could not be considered heritable as a unit. Altogether, these results reinforce the idea that the mechanisms for coral–microbial symbiosis could be more flexible and dynamic than first thought (Boulotte et al., 2016).

Not only are the symbiotic mechanisms not yet fully understood, but there is also a great paucity of knowledge on the potential beneficial traits of microbial holobiont members that are vertically or horizontally transferred during spawning and this remains an important issue for ongoing studies. Antimicrobial activity of symbiont microbes may serve as a protective function for coral spawned gametes or larvae. As noted by Marquis et al. (2005), *Montipora digitata* seeds its eggs inside bundles with *Symbiodinium*, which might suggest a potentially protective role for *Symbiodinium*-derived metabolites. A recent study of Lin et al. (2015) found biochemical complementarity between the genomes of the symbiont and the host, which is indicative of coevolution. These data seem to underpin the need to guarantee the transfer of the microbiota, even if complementary strategies of transmission are needed. Most studies have indicated *Roseobacter* and *Alteromonas* as genera that occur in the early stages of coral life (Sharp et al., 2011; Ceh et al., 2012, 2013). However, we found here that at least six genera are associated with these stages, with *Burkholderia* sp. being the most abundant genus (approximately 75–85%). Recent studies have suggested that members of this genus are also vertically transmitted in plants (*Sphagnum* spp.) (Bragina et al., 2013). Several *Burkholderia* species are capable of fixing nitrogen and degrading aromatic compounds (Suárez-Moreno et al., 2012), whereas others strongly interact with fungi and/or cold-adapted plants. The presence of N-fixing bacteria was previously observed in the initial stages of coral life (Lema et al., 2014). Thus, bacteria can potentially benefit the coral host, since these features seem to be important to coral symbionts (Santos et al., 2014) and to the protection of coral from environmental impact (Santos et al., 2015).

Some studies have described a *Pseudomonas* sp. isolated from corals with antimicrobial and anti-pathogenic potentials, which could be important to regulate the presence of some microorganism or prevent the establishment of pathogens during early stages of coral life (Radjasa et al., 2007; Bakkiyaraj et al., 2013). Members of the genus *Pseudomonas* are also genetically very plastic, including different abilities that are important to corals, such us nitrogen fixation, pollutant degradation, use of different carbon sources to provide nutrients and production of antibiotics (Garcia-Valdes et al., 1988; Das and Mukherjee, 2007; Rojo, 2010). These features seem to provide clues about the importance of this genus being transmitted to coral offspring.

Corals from the Southwestern Atlantic are located in waters with higher sedimentation rates compared to those of the Pacific Ocean (Ricardo et al., 2015). Suspended sediment can represent a risk to the reproductive processes of corals because high sediment concentrations in suspension are capable of removing sperm from the seawater surface during reproduction events (Ricardo et al., 2015). This would decrease the contact time between the male and female gametes and might compromise recruitment rates and, in turn, have a negative population effect (Ricardo et al., 2015). We believe that corals from the Brazilian coast have developed specific strategies to live and survive in turbid waters. In its embryonic and planula larvae stages, the coral *M. hispida*, besides being able to transfer *Symbiodinium* cells to its offspring, also hosts bacterial genera that have not yet been described as part of the microbiome of other coral species.

The specific microorganisms transmitted by coral from generation to generation are assumed to be key to the sustenance of the coral life. It is likely that such transmission reflects some level of selection and influences host fitness. The HTE hypothesizes that the holobiont is the unit of selection in evolution and that any genetic variation in the holobiont, occurring in the host and/or in the microbial symbiont genomes, in cases of selective advantage is successfully transmitted to the offspring. This theory places as much emphasis on cooperation as it does on competition according to the principle of ‘survival of the fittest’ or as a result of stochastic events (Rosenberg et al., 2009). Even though our data indicate VT in *M. hispida*, we agree that this is not enough to assume co-evolution, as stated by other authors, including Hester et al. (2016), although it can be considered as additional evidence. More data are required to indicate whether the stable transmitted populations and the hosts are indeed units of natural selection or symbiotic assemblages of independently evolving organisms. The organisms that constitute the holobiont need to fit together in a tight relationship (including the vertical microbiome transmission demonstrated here in *M. hispida*) so that the holobiont adapts to the environment.

## Conclusion

The spawner coral *M. hispida* can transfer its bacterial and *Symbiodinium* cells to its offspring through VT. The core bacterial populations found in the bundles, planula larvae and parental colonies were identified as members of the genera *Burkholderia, Pseudomonas, Acinetobacter, Ralstonia, Inquilinus*, and *Bacillus*. The abundance of these populations was shown to be dynamic and related to coral life stages. While the stable microbiome represents a greater proportion of the associated bacteria in embryonic and planula larvae stages, the core microbiome represents a low proportion in the adult colonies. This result suggests that *M. hispida* has parallel and different strategies for its microbiome acquisition. We hypothesized that coral offspring can be partly post-spawning colonized through the uptake of microbial associates released by the parental colony into the surrounding seawater and partly colonized by parental colonies, which transfer their bacterial core and *Symbiodinium* cells by seeding the mucus and the gametes, respectively. Being dynamic, the coral holobiont may incorporate part of its associated microbiota from the water column every time an adaptation to new environmental conditions is required, and this plasticity and capacity to modulate the microbiome uptake could be an important strategy of corals that could explain their billions of years ancient existence and persistence.

## Author Contributions

Study conception and design: RP, AR, DL, HS, CC, DP. Acquisition and identification of coral samples: DL, CC, DP. Acquisition of data (experimental development): DL, PL, UL, AG, CZ. Analyses and interpretation of data: RP, AR, DL, PL, UL, AG, JvE, CZ, CC, DP. Drafting of the manuscript: RP, DL. Critical revision: all authors. Financial support: RP, AR, UL, CC, CZ.

## Conflict of Interest Statement

The authors declare that the research was conducted in the absence of any commercial or financial relationships that could be construed as a potential conflict of interest.

## Footnotes

1 http://blast.ncbi.nlm.nih.gov/Blast.cgi

2 http://tree.bio.ed.ac.uk/software/figtree/

## Supplementary Material

FIGURE S1 **Scheme showing the experimental strategy used in this study.** Boxes in gray scale: collected samples are represented from embryonic stage (bundles, spawning time) to coral planula larvae (3 and 5 days a.f.). Blue rectangles: techniques used in this study; bacterial communities profile and composition were evaluated by PCR-DGGE and sequencing, respectively, and zooxanthellae diversity was evaluated by PCR and cloning/sequencing.

FIGURE S2 **Richness indexes for bacterial communities associated with different life stages of *Mussismilia hispida* and surrounding seawater. (A)** Phylogenetic distance, **(B)** Chao and **(C)** Observed OTUs. W0d: seawater at the spawning event, W5d: seawater 5 days after the spawning event, G0d: Bundles at the spawning event, L5d: coral planula larvae, 5 days a.f., and MH: adult colonies of *M. hispida*.

FIGURE S3 **Relative abundance of the variable and core microbiome from different *M. hispida* life stages, according to 16S rRNA gene high throughput (Ion Torrent PGM) sequencing data.** G0d: bundles at the spawning event, L5d: coral planula larvae, 5 days a.f., and MH: adult colonies of *M. hispida*.

FIGURE S4 **Light microscopy micrograph of a thin section of *M. hispida* bundles, highlighting male and female gametes.** F: female gametes. M: spermatic cysts.

TABLE S1 **Permutational statistical (PERMANOVA) analyses of DGGE and sequencing data to assess the water and *Mussismilia hispida* microbiome**.

TABLE S2 **Bacterial core community of different life stages of *M. hispida* at OTU level.** G0d: bundles at the spawning event, L5d: coral planula larvae, 5 days a.f., and MH: adult colonies of *M. hispida*.
