# Supplementary figures and images for: Broadcast Spawning Coral Mussismilia hispida Can Vertically Transfer its Associated Bacterial Core
Source: Front Microbiol. 2017 Feb 7;8:176. doi: 10.3389/fmicb.2017.00176 (PMC5293827; doi:10.3389/fmicb.2017.00176)

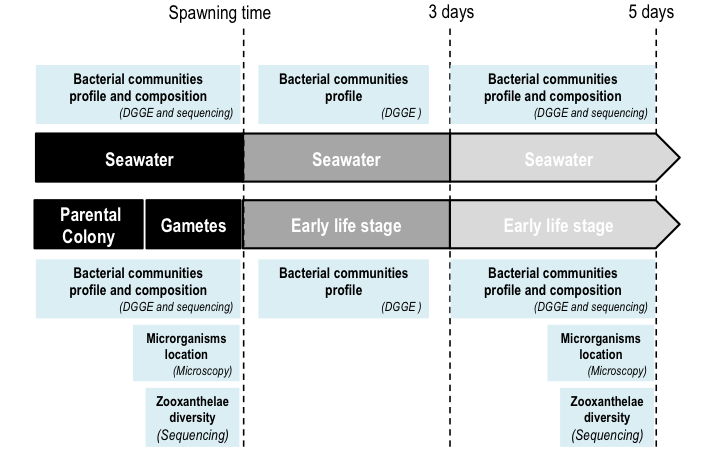

Supplement: FIGURE S1 — Scheme showing the experimental strategy used in this study. Boxes in gray scale: collected samples are represented from embryonic stage (bundles, spawning time) to coral planula larvae (3 and 5 days a.f.). Blue rectangles: techniques used in this study; bacterial communities profile and composition were evaluated by PCR-DGGE and sequencing, respectively, and zooxanthellae diversity was evaluated by PCR and cloning/sequencing. [file Image_1.TIFF]

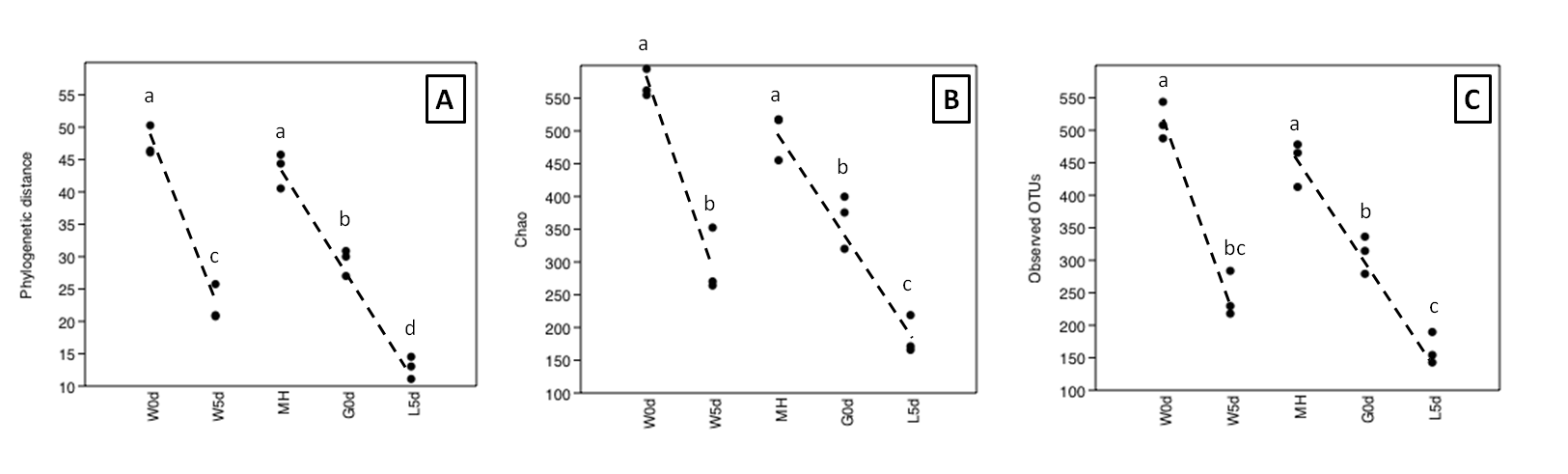

Supplement: FIGURE S2 — Richness indexes for bacterial communities associated with different life stages of Mussismilia hispida and surrounding seawater. (A) Phylogenetic distance, (B) Chao and (C) Observed OTUs. W0d: seawater at the spawning event, W5d: seawater 5 days after the spawning event, G0d: Bundles at the spawning event, L5d: coral planula larvae, 5 days a.f., and MH: adult colonies of M. hispida. [file Image_2.TIFF]

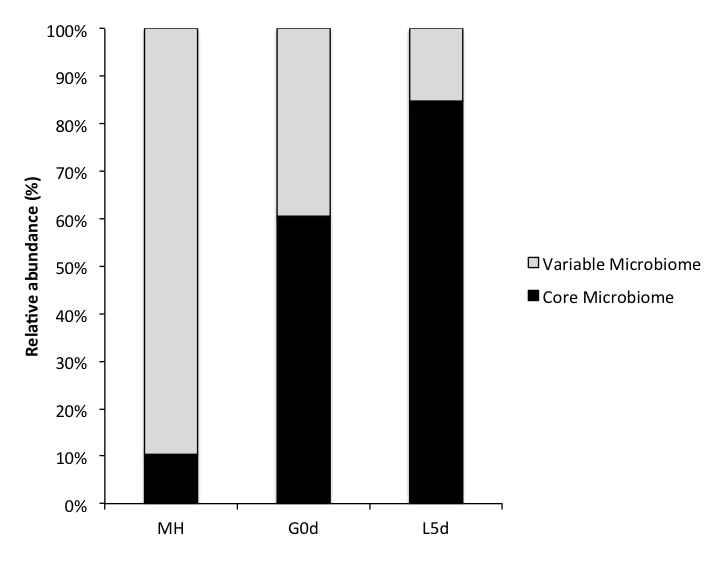

Supplement: FIGURE S3 — Relative abundance of the variable and core microbiome from different M. hispida life stages, according to 16S rRNA gene high throughput (Ion Torrent PGM) sequencing data. G0d: bundles at the spawning event, L5d: coral planula larvae, 5 days a.f., and MH: adult colonies of M. hispida. [file Image_3.TIFF]

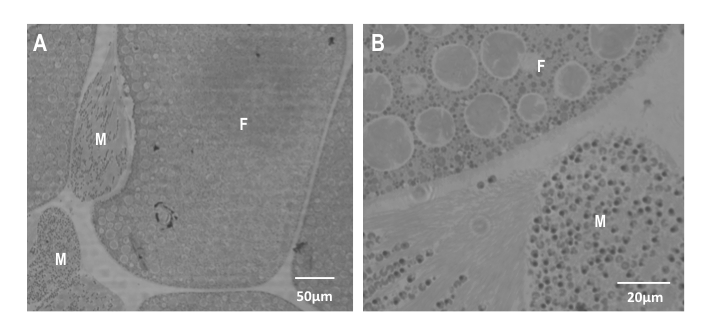

Supplement: FIGURE S4 — Light microscopy micrograph of a thin section of M. hispida bundles, highlighting male and female gametes. F: female gametes. M: spermatic cysts. [file Image_4.TIFF]
