# Supplementary material for: Broadcast Spawning Coral Mussismilia hispida Can Vertically Transfer its Associated Bacterial Core
Source: Front Microbiol. 2017 Feb 7;8:176. doi: 10.3389/fmicb.2017.00176 (PMC5293827; doi:10.3389/fmicb.2017.00176)
Supplement: TABLE S1 — Permutational statistical (PERMANOVA) analyses of DGGE and sequencing data to assess the water and Mussismilia hispida microbiome. [file Table_1.DOCX]

Table S1

| **Study method and** | **PERMANOVA** | |
| --- | --- | --- |
| **Variable** | **t** | ***P*(perm)** |
| *DGGE* |  |  |
| Coral x Gamete | 1.76 | 0.09 |
| Coral X Coral early life stage | 2.26 | **0.01** |
| Gamete x Coral early life stage | 2.94 | **0.01** |
| Gamete x Water | 2.57 | **< 0.01** |
| Coral early life stage x Water | 3.67 | **< 0.01** |
| Coral x Water | 1.80 | **< 0.01** |
| *Sequencing* |  |  |
| Coral x Gamete | 7.66 | 0.10 |
| Coral X Coral early life stage | 7.21 | 0.10 |
| Gamete x Coral early life stage | 3.39 | 0.10 |
| Gamete x Water | 2.27 | **0.03** |
| Coral early life stage x Water | 2.38 | **0.02** |
| Coral x Water | 2.57 | **0.03** |
