# Supplementary material for: Broadcast Spawning Coral Mussismilia hispida Can Vertically Transfer its Associated Bacterial Core
Source: Front Microbiol. 2017 Feb 7;8:176. doi: 10.3389/fmicb.2017.00176 (PMC5293827; doi:10.3389/fmicb.2017.00176)
Supplement: TABLE S2 — Bacterial core community of different life stages of M. hispida at OTU level. G0d: bundles at the spawning event, L5d: coral planula larvae, 5 days a.f., and MH: adult colonies of M. hispida. [file Table_2.DOCX]

Table S2
